# Methimazole‐induced cholestasis initially obscured by an incidental finding of a large periampullary diverticulum: A case report with reference to the updated RUCAM of 2016

**DOI:** 10.1002/jgh3.13042

**Published:** 2025-02-24

**Authors:** Salomon Chamay, Ari Alter, Rooshi Parikh, Shivam Khatri, Deepa Budh, Mitchell Spinnell

**Affiliations:** ^1^ Division of Internal Medicine, Department of Medicine St. Barnabas Hospital Bronx New York USA; ^2^ Touro University New York New York USA; ^3^ CUNY School of Medicine New York New York USA; ^4^ Division of Gastroenterology, Department of Medicine Englewood Hospital Englewood New Jersey USA

**Keywords:** cholestatic liver injury, drug‐induced liver injury, methimazole‐induced liver injury, obstructive jaundice, periampullary diverticulosis, updated RUCAM

## Abstract

Methimazole is a commonly prescribed drug for hyperthyroidism and is generally well‐tolerated, with complications occurring in less than 10% of treated patients. To our knowledge, there are approximately 30 reported cases of drug‐induced liver injury (DILI) associated with the use of methimazole in the literature. DILI is a challenging diagnosis and can often mimic many forms of acute and chronic hepatitis. The pattern of liver injury can be hepatocellular, cholestatic, or mixed in nature. The R‐value is a useful tool for characterizing the pattern of liver injury, while the Roussel Uclaf Causality Assessment Method (RUCAM) aids with quantifying the likelihood of DILI. The updated version of RUCAM from 2016 was used in this report. We present a case of a 72‐year‐old female who developed cholestatic DILI secondary to methimazole with a calculated RUCAM score of 8, initially obscured by both imaging and endoscopic findings concerning for hepatobiliary obstruction.

## Abstract 2

Methimazole is a commonly prescribed drug for hyperthyroidsm with a low incidence of adverse side effects. There are very few reports of drug‐induced liver injury (DILI) secondary to methimazole found within the literatrue. We present a case of a 72‐year‐old female who developed cholestatic DILI secondary to methimazole initially obscured by both imaging and endoscopic findings concerning for hepatobiliary obstruction.

## Introduction

Drug‐induced liver injury (DILI) is a rare and challenging medical condition that can be classified as direct or idiosyncratic. Idiosyncratic DILI is caused by agents with little to no intrinsic toxicity and is further categorized as hepatocellular, cholestatic, or mixed in nature on the basis of the R ratio, a relationship between alanine aminotransferase (ALT) and alkaline phosphatase (ALP) levels. It has an estimated incidence of 14–19 cases per 100 000 persons each year and is the most frequent cause of acute liver failure in most Western countries.^1^ The diagnosis of DILI is one of the exclusion criteria and requires a high index of clinical suspicion, as its presentation can mimic any form of acute and chronic hepatobiliary disease.^2^, ^3^ Methimazole, a common therapeutic agent for hyperthyroidism, is generally well‐tolerated, with complications occurring in less than 10% of treated patients.^4^ Of those complications, DILI has been described in extremely rare instances.

## Case presentation

A 72‐year‐old female with a medical history of hyperlipidemia, vertigo, spinal stenosis, and prior cerebrovascular accident presented with a chief complaint of abdominal pain for two weeks associated with jaundice, acolia, dark urine, pruritus, nausea, and a 10‐lb weight loss. The patient was diagnosed with hyperthyroidism 8 weeks prior to presentation, characterized by persistently low TSH, anxiety, tremors, and weight loss. She was started on methimazole 5 mg daily. Her home medications included meclizine 25 mg daily as needed and aspirin 81 mg daily. Her social history was notable for minimal tobacco consumption, equivalent to six pack‐years. Physical examination was remarkable for right upper quadrant abdominal pain without rebound tenderness or guarding, and jaundice of the skin and sclera. Vital signs were within normal limits.

Initial laboratory testing revealed transaminitis along with elevated total bilirubin and ALP (Table 1).

**Table 1:** Patient's liver function testing (LFT) at the time of her presentation until resolution

Laboratory findings **(**Table 1**)**					
	03/30/2022	04/15/2022	05/09/2022	07/2022	Normal value
AST	326 U/L	165 U/L	36 U/L	18 U/L	13–39 U/L
ALT	237 U/L	125 U/L	23 U/L	7 U/L	7–52 U/L
Alkaline phosphatase	1052 U/L	1032 U/L	340 U/L	76 U/L	44–147 U/L
Bilirubin total	5.6 mg/dL	10.0 mg/dL	2.2 mg/dL	0.4 mg/dL	0.3–1.0 mg/dL
Direct bilirubin	3.2 mg/dL	6.8 mg/dL	N/A	N/A	<0.3 mg/dL
Indirect bilirubin	2.4 mg/dL	3.2 mg/dL	N/A	N/A	0.2–0.8 mg/dL

CBD stent was placed on 3/31/2022. Methimazole was discontinued on 4/5/2022.

An abdominal ultrasound reported biliary dilation without the presence of gallstones. Abdominal computed tomography scan (CT) showed proximal bile duct dilation with distal narrowing at the porta hepatis, a duodenal diverticulum, and no evidence of obstructing stone or mass. The gallbladder and pancreas were unremarkable. Endoscopic retrograde cholangiopancreatography (ERCP) was performed and revealed a large periampullary diverticulum in the second portion of the duodenum, and a stricture measuring approximately 4 cm in length in the distal common bile duct (CBD), extending into the intrapancreatic portion. Above the stricture, the bile duct measured 14 mm with additional dilatation of the gallbladder and intrahepatic ducts. Sphincterotomy was performed, and brushings were taken from the distal bile duct to rule out malignancy. A fully covered metal stent was placed across the CBD stricture.

Initial brushings were negative for malignancy. Therefore, endoscopic ultrasound (EUS) was performed, revealing thickening of the bile duct wall in the distal segment. Large periportal lymphadenopathy measuring 1.5–1.8 cm in size was also noted on EUS and sampled using fine‐needle aspiration (FNA). The inspected portions of the liver showed no signs of metastatic disease.

FNA results remained non‐diagnostic; therefore, a second technique using choledochoscopy was considered. At this point in time, laboratory studies to assess for viral and autoimmune hepatic disease were negative. Cytology brushings from ERCP and EUS returned negative.

Prior to undergoing a second ERCP, methimazole was stopped due to concern for DILI. The patient's calculated R‐value was 0.64, suggestive of cholestatic pattern, and her calculated RUCAM score of 8 indicated an increased likelihood for DILI. Two days after discontinuation of methimazole, the patient's symptoms improved. A second ERCP with choledochoscopy spyglass was performed, which showed a narrowed diameter of the bile duct and normal bile duct mucosa. Additionally, biopsies confirmed normal bile duct mucosa and were negative for malignancy. A week after the procedure and discontinuation of methimazole, the patient's symptoms resolved, and her LFTs began trending down to normal values over the next several months. Shortly after normalization of her LFTs, the CBD stent was removed, without recurrence of symptoms. At her most recent follow‐up 6 months later, the patient was completely asymptomatic and her LFTs remained normal.

## Discussion

DILI is a rare, often challenging entity faced by many clinicians. It occurs with an estimated incidence of 14–19 cases per 100 000 persons, is responsible for 3–5% of hospital admissions for jaundice, and is the most common cause of acute liver failure in most Western countries.^1^ Although large advances have been made in the field of hepatology, the diagnosis of DILI still relies heavily on the exclusion of other etiologies.^1^, ^2^, ^3^ Key diagnostic features are latency of injury, resolution after discontinuation of the offending agent, recurrence on re‐exposure or rechallenge, knowledge of the agent's hepatotoxic potential, and phenotypic features.^1^

The hepatotoxicity of DILI can be classified as either direct, when the agent is known to be intrinsically toxic to the liver, or idiosyncratic when the agent has little or no intrinsic toxicity. In contrast to direct DILI, idiosyncratic hepatotoxicity is unpredictable, rare, and not dose‐dependent.^1^ The R ratio, a relationship between alanine aminotransferase and ALP levels from the time of initial presentation, further categorizes idiosyncratic DILI into hepatocellular, cholestatic, or mixed in nature.^5^ An R‐value greater than 5, less than 2, and between 2 and 5 define liver injury as hepatocellular, cholestatic, or mixed, respectively.^5^

Cholestatic DILI is usually self‐limited in nature and presents predominantly as symptoms of pruritus and jaundice accompanied by moderate to high elevations of ALP.^1^ Other initial presentations described include acute abdominal pain and fever.^2^ It is hypothesized that the cholestatic pattern may be caused by canalicular cholestasis or ductular injury.^2^ Additionally, although cholestatic DILI can lead to chronic ductopenia and rarely cirrhosis, it is not as life‐threatening as hepatocellular DILI.^2^ Common drugs associated with a cholestatic pattern include amoxicillin‐clavulanate, cephalosporins, erythromycin, terbinafine, azathioprine, and temozolomide.^1^, ^3^

The Roussel Uclaf Causality Assessment Method (RUCAM) score is an assessment tool utilized to quantify the likelihood of hepatic injury due to a specific drug. It takes into consideration the time of onset, course, risk factors, concomitant drugs, nondrug causes, previous information on the hepatotoxic profile of the drug, and the response to rechallenge. It is currently regarded as one of the best diagnostic tools available to clinicians for causality assessment of DILI.^1^, ^2^, ^5^, ^6^ RUCAM's importance is highlighted by the fact that it has been widely adopted and utilized for evaluating the causality of DILI cases. As of mid‐2020, a staggering 81 856 cases of DILI have been published and documented worldwide either referencing the original RUCAM from 1993 or the updated 2016 version, with each case meticulously assessed for causality using RUCAM.^7^ The comprehensive nature of RUCAM enables medical professionals to assess the relationship between the timing of drug exposure, the clinical presentation of liver injury, and any potential alternative explanations for the hepatotoxicity.

In the literature, there have been approximately 30 reported cases of methimazole‐induced hepatotoxicity.^8^ Ji *et al*. presented the case of a 69‐year‐old male being treated with methimazole for hyperthyroidism and carbamazepine for epilepsy. The authors used the RUCAM scale to diagnose methimazole‐induced cholestatic jaundice.^8^ Li *et al*. compared 40 patients with methimazole‐induced liver injury to 118 control patients taking methimazole without an evidence of liver injury and found the HLA‐C 03:02 allele was associated with an increased risk of developing methimazole‐induced hepatic injury. The authors used the RUCAM score to confirm methimazole as the most likely cause of hepatic injury in the 40‐patient group.^9^ Zou *et al*. presented the cases of a 50‐year‐old female and 42‐year‐old female with a history of hyperthyroidism treated with methimazole later causing cholestatic jaundice. The authors did not use the RUCAM scale, recommending proper patient education and immediate cessation of methimazole if symptoms of hepatotoxicity occur.^4^ Similarly, Abramavicius *et al*. presented the case of 74‐year‐old female being treated with methylprednisone and methimazole for thyrotoxicosis and Graves ophthalmopathy, later complicated by DILI. The authors did not use the RUCAM scale, stating continuation of methylprednisone and a lowered dose of methimazole still be used as treatment if no other options are available.^10^

Females are predominantly affected by drug‐induced hepatotoxicity and have a higher incidence of hyperthyroidism.^11^ The reported latency period for methimazole‐induced cholestasis ranges from 2 days to 3 months, and as with most cholestatic DILI presentations, the outcome is usually benign.^4^ Resolution of DILI following discontinuation of methimazole has been reported to occur within 5 days to 6 months.^4^

In our case, the patient presented with clear signs and symptoms of cholestatic disease. Both clinically and radiographically, the patient's presentation raised concern for obstructive pathology within the biliary tree. Additionally, endoscopic evaluation demonstrated evidence of a stricture with distal narrowing within the CBD and significant proximal dilation up to 14 mm. Initial sphincterotomy and stenting, however, did not resolve the patient's symptoms and cytology resulted negative for occult malignancy within the biliary tree. Moreover, autoimmune and infectious etiologies were ruled out. Further endoscopic evaluation using choledochoscopy spyglass showed normal bile duct mucosa within the narrowing. Alternate causes of cholestasis were considered, and the patient's *R*‐value at the time of symptom onset was calculated to be 0.64, which was consistent with a cholestatic pattern of liver injury. Additionally, her RUCAM calculation yielded a score of 8 points, further increasing the likelihood for DILI. The temporal relationship between initiation of methimazole and onset of symptoms further argued in favor of DILI. Upon discontinuation of methimazole, the patient's symptoms began to improve, and this was supported by a downward trend of her LFTs. After normalization of LFTs, the CBD stent was removed, and the patient remained asymptomatic. Additionally, drainage from the bile duct was noted even after the removal of the stent. Six months after discontinuation of methimazole and removal of the CBD stent, her LFTs remained normal, and the patient was symptom free.

This case serves to highlight the high degree of difficulty that exists with diagnosing DILI. It has been described in the literature that DILI can mimic many forms of acute and chronic hepatitis. In our case, the patient presented with clear signs concerning hepatobiliary obstruction. DILI should be considered in the differential diagnosis when evaluating a patient with cholestasis and obstructive symptomatology.

## Conclusion

DILI is a rare and clinically challenging form of liver disease that requires careful consideration and exclusion of other disease processes. It may mimic all forms of acute and chronic hepatitis. When a patient presents with jaundice, abdominal pain, and concern for hepatobiliary obstruction, a diagnosis of cholestatic DILI should be considered in the differential.

## Patient consent

Written informed consent was obtained from the patient for publication and any accompanying images. A copy of the written consent is available for review by the Editor‐in‐Chief of this journal on request.

## Ethics approval

Ethics approval was waived by the Englewood Hospital as case reports do not require ethics approval at our hospital.

## Patient consent

Written informed consent was obtained from the patient for publication and any accompanying images. A copy of the written consent is available for review by the Editor‐in‐Chief of this journal on request.

## Data Availability

All data is available on request.
